# Supplementary material for: ChromNet: Learning the human chromatin network from all ENCODE ChIP-seq data
Source: Genome Biol. 2016 Apr 30;17:82. doi: 10.1186/s13059-016-0925-0 (PMC4852466; doi:10.1186/s13059-016-0925-0)
Supplement: Additional file 1 — Supplementary information. (PDF 4,962 kb) [file 13059_2016_925_MOESM1_ESM.pdf]

ChromNet: Learning the human chromatin network from all ENCODE ChIP-seq data

Scott M. Lundberg[1], William B. Tu[2,3],
Brian Raught[2,3], Linda Z. Penn[2,3], Michael M. Hoffman[2,3,4], Su-In Lee[1,5]

[1] Department of Computer Science and Engineering, University of Washington
[2] Department of Medical Biophysics, University of Toronto
[3] Princess Margaret Cancer Centre
[4] Department of Computer Science, University of Toronto
[5] Department of Genome Sciences, University of Washington

Supplementary information

Supplementary figures

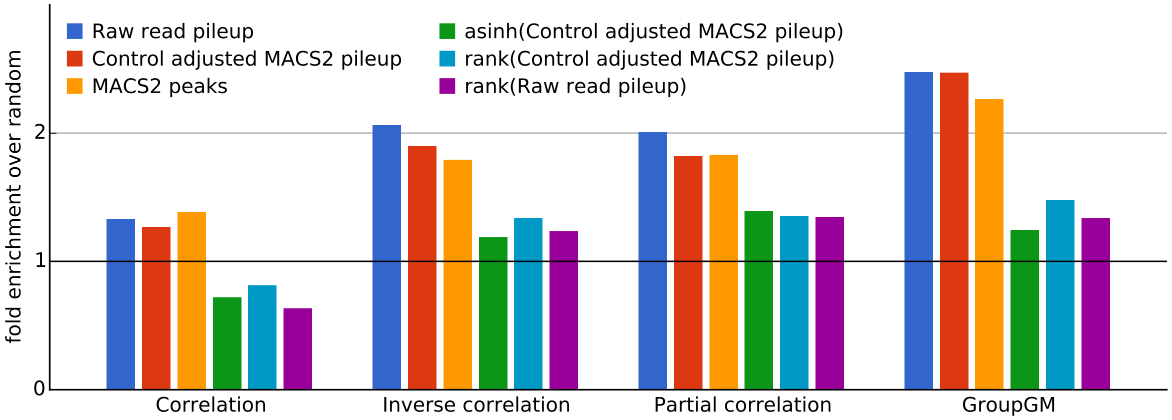

Figure S1: Enrichment of BioGRID-supported edges within all cell lines across four different modeling approaches and six different pre-processing methods. For raw pileup (blue) we binned raw Hg38 mapped read start sites. For control-adjusted pileup (red), we took MACS2 pileup output and normalized by a paired control. For MACS2 peaks (yellow), we used MACS2 with paired controls and a lenient peak threshold (varying the threshold produced similar results, see Figure S2). For each of the data transforms we applied the given function to the value in each 1,000 bp bin.

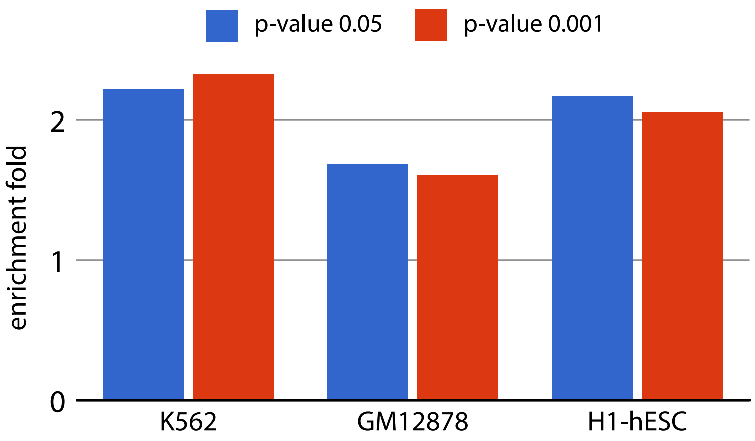

Figure S2: Enrichment of BioGRID-supported edges in a GroupGM created from a binary data matrix of MACS peaks called at two different thresholds ($P < 0.05$, blue; $P < 0.001$, red). Within the larger network we examined BioGRID enrichment among ENCODE tier 1 cell lines: K562 myeloid leukemia cells, GM12878 lymphoblastoid cells, and H1-hESC embryonic stem cells.

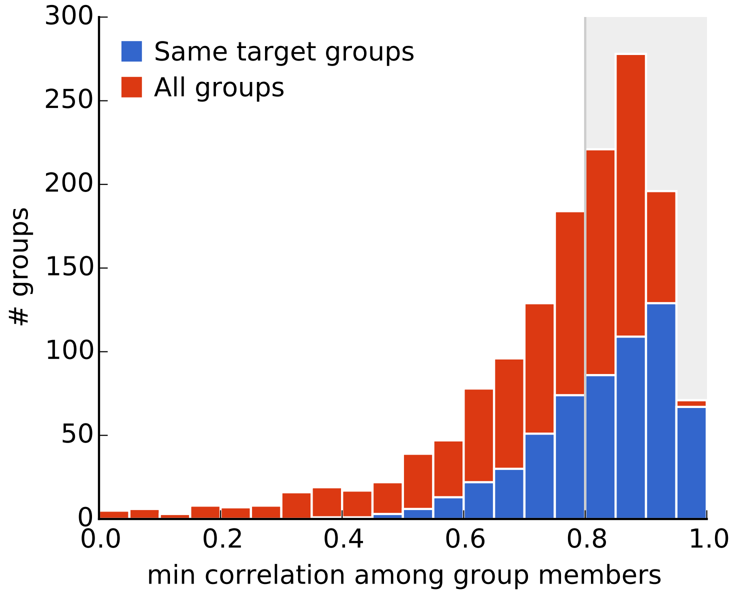

Figure S3: Distribution of group widths within ChromNet. Groups containing only a single regulatory factor type tend to have a stronger correlation, but many heterogeneous groups also show tight correlations. The gray region highlights the groups we allowed in the ChromNet network used for analysis in this paper.

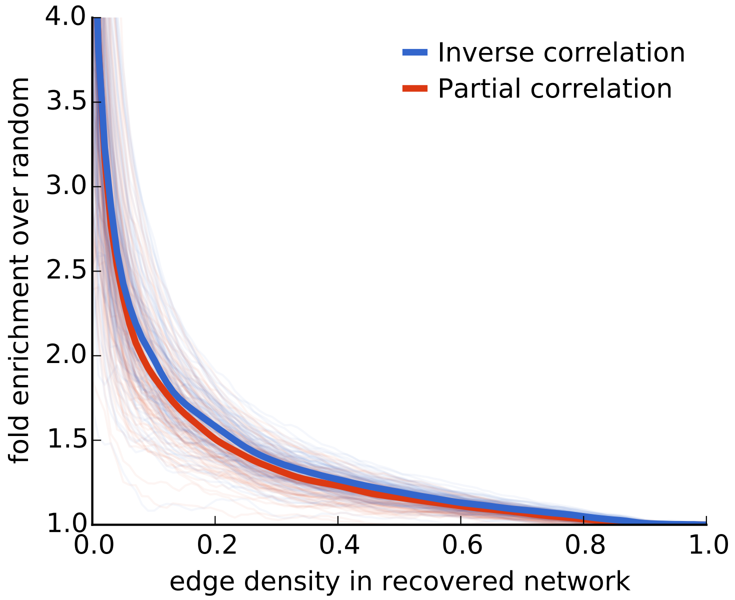

Figure S4: Comparison of BioGRID enrichment performance between inverse correlation and partial correlation. Partial correlation can be viewed as a re-normalized version of the inverse correlation matrix, but is not used in ChromNet since the group graphical model proof is specific to the inverse correlation matrix.

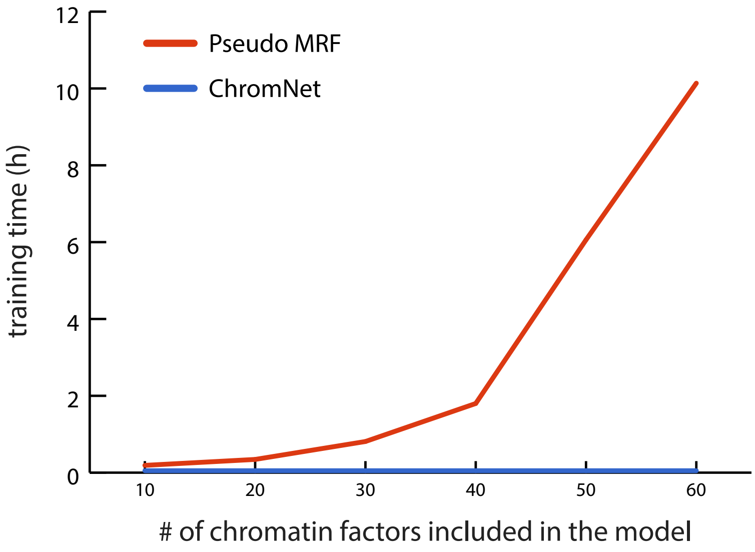

Figure S5: Wall clock training time to fit the pairwise pseudo-likelihood Markov random field model from Zhou et al. [14] on ENCODE data. As the number of variables in the model increases, the method's running time becomes infeasible. We tuned regularization parameters using the same 61 warm-started optimizations used in [14]. We ran this test on a 12-core Intel Xeon CPU E5645 2.40GHz computer with 24 GB of random access memory.

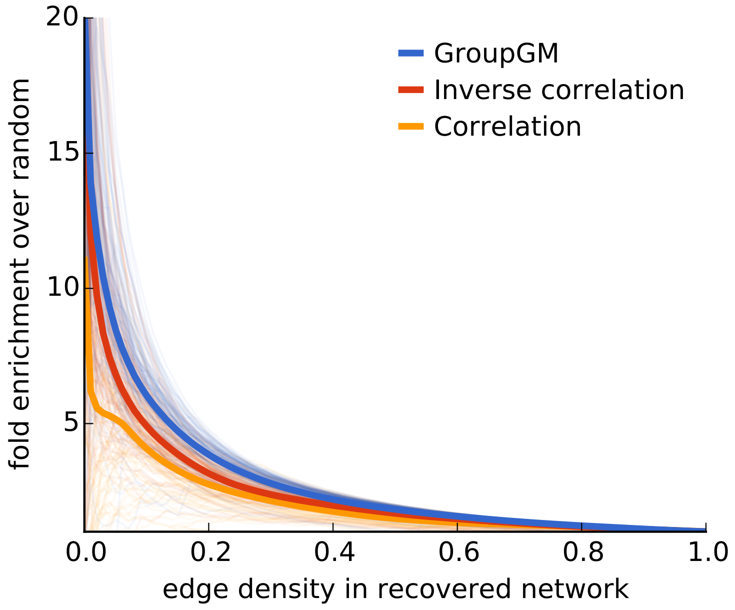

Figure S6: Just as BioGRID was used to validate the performance of protein-protein connections in ChromNet (Figure 3A), histone mark writers can be used to validate protein/histone-mark connections. All histone-mark/writer combinations were taken from the HIstome database [4] and enrichment for these edges among all protein/histone-mark connections was calculated.

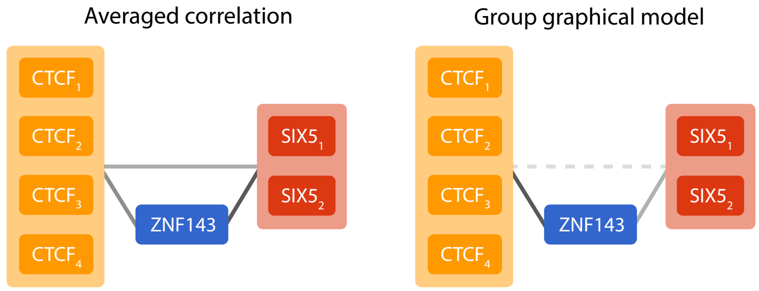

Figure S7: The factors CTCF and SIX5 closely associate specifically when ZNF143 is also present. This causes ZNF143 to mediate the interaction between CTCF and SIX5. The presence of ZNF143 can be also be viewed as the "context" in which CTCF and SIX5 co-localize (Figure S19).

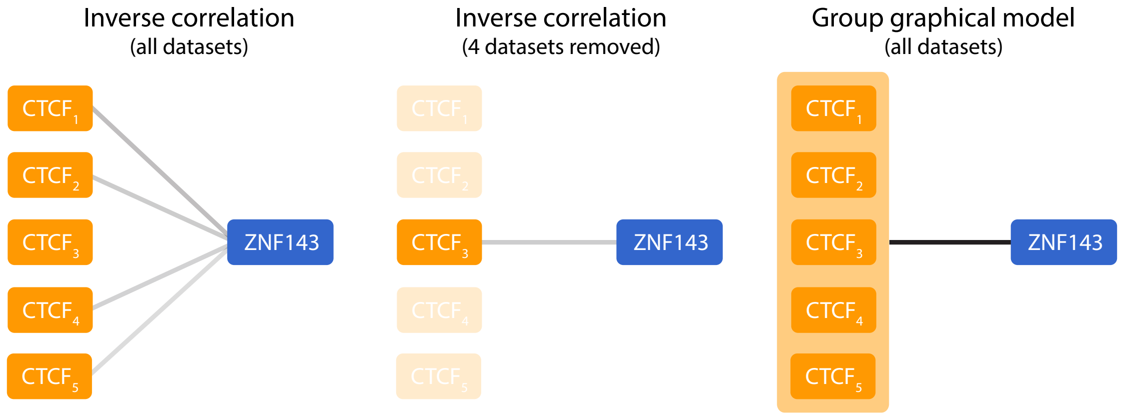

Figure S8: Illustration of how GroupGM helps with problems in inverse correlation caused by collinearity. When using all 1,451 datasets inverse correlation recovers edges between ZNF143 and four of the five CTCF datasets in K562 *(left)*. When the four datasets with an edge to ZNF143 are removed the other dataset gets an edge to ZNF143 stronger than any of the original four datasets *(middle)*. This means that redundancy with the other datasets caused the other dataset to be ignored, even though it was strongly related to ZNF143. In contrast the group graphical model recovers a much stronger edge between CTCF and ZNF143 *(right)*.

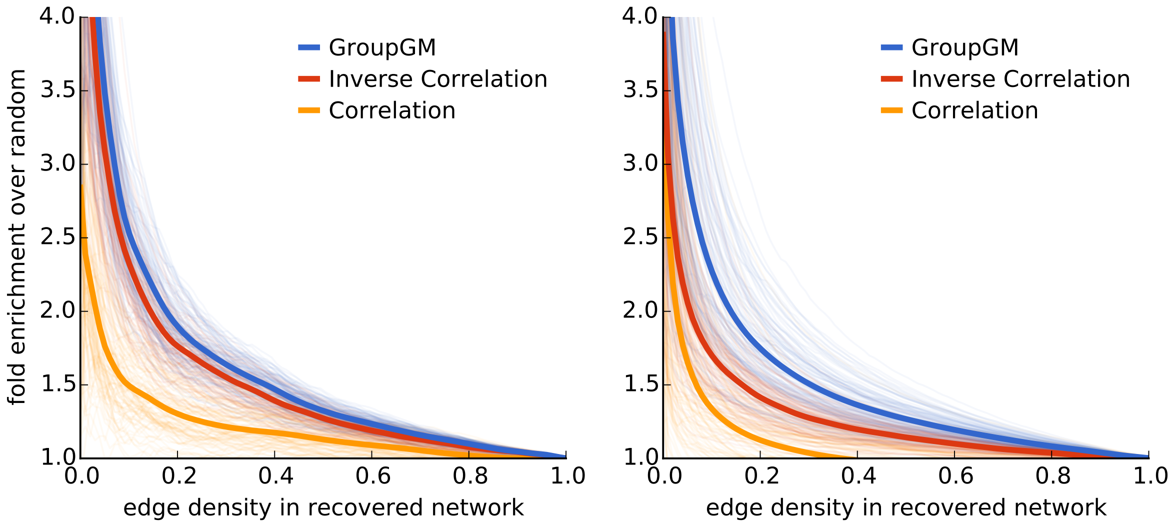

Figure S9: Some datasets target the same factor in the same cell type/condition. Here we average those datasets under the assumption that the distinction between them is not important. GroupGM still provides an improvement even in the absence of these potentially redundant datasets both within cell types (P-value = 0.002) and between cell types (P-value = 0.021). The left figure is enrichment for BioGRID supported edges within all cell types, while the right figure is enrichment between all cell types.

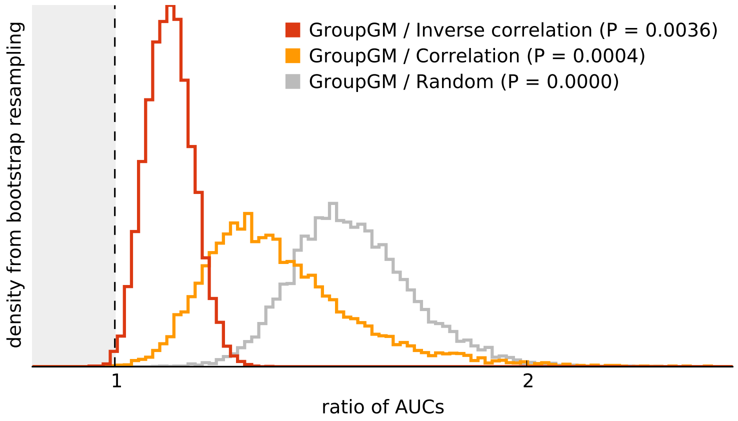

Figure S10: Histogram of area under the curve (AUC) ratios comparing enrichment of BioGRID-supported edges in a GroupGM network versus networks created by inverse correlation (red), correlation (yellow), and random edge score assignment (grey). Specifically, we compared the area under enrichment–edge density curves from 10,000 bootstrap samples from regulatory factors, excluding edges between different cell types (Figure 3A top). P-values represent the fraction of bootstrap samples with a ratio of AUC's less than 1. Being less than 1 means that GroupGM performed worse than the alternative method.

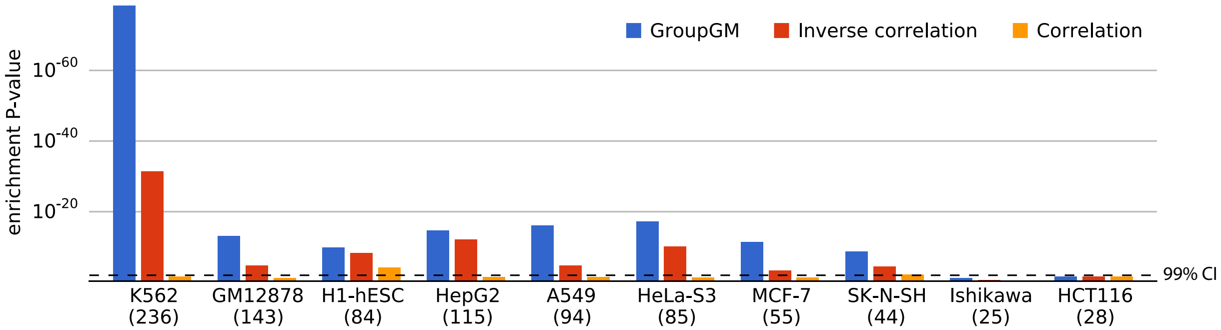

Figure S11: One-sided hypergeometric test negative $\log_{10}$ P-values for enrichment of BioGRID-supported edges within cell types that have 25 supported edges or more (Figure 3C). The hypergeometric test is less conservative than the bootstrap approach used in Figure S10 and Figure S14. Cell types with more datasets will likely have more significant P-values, since they have more edges to compare. Dashed line indicates 99% confidence level ($P = 0.01$). Beneath each cell type name is the number of datasets in that cell type.

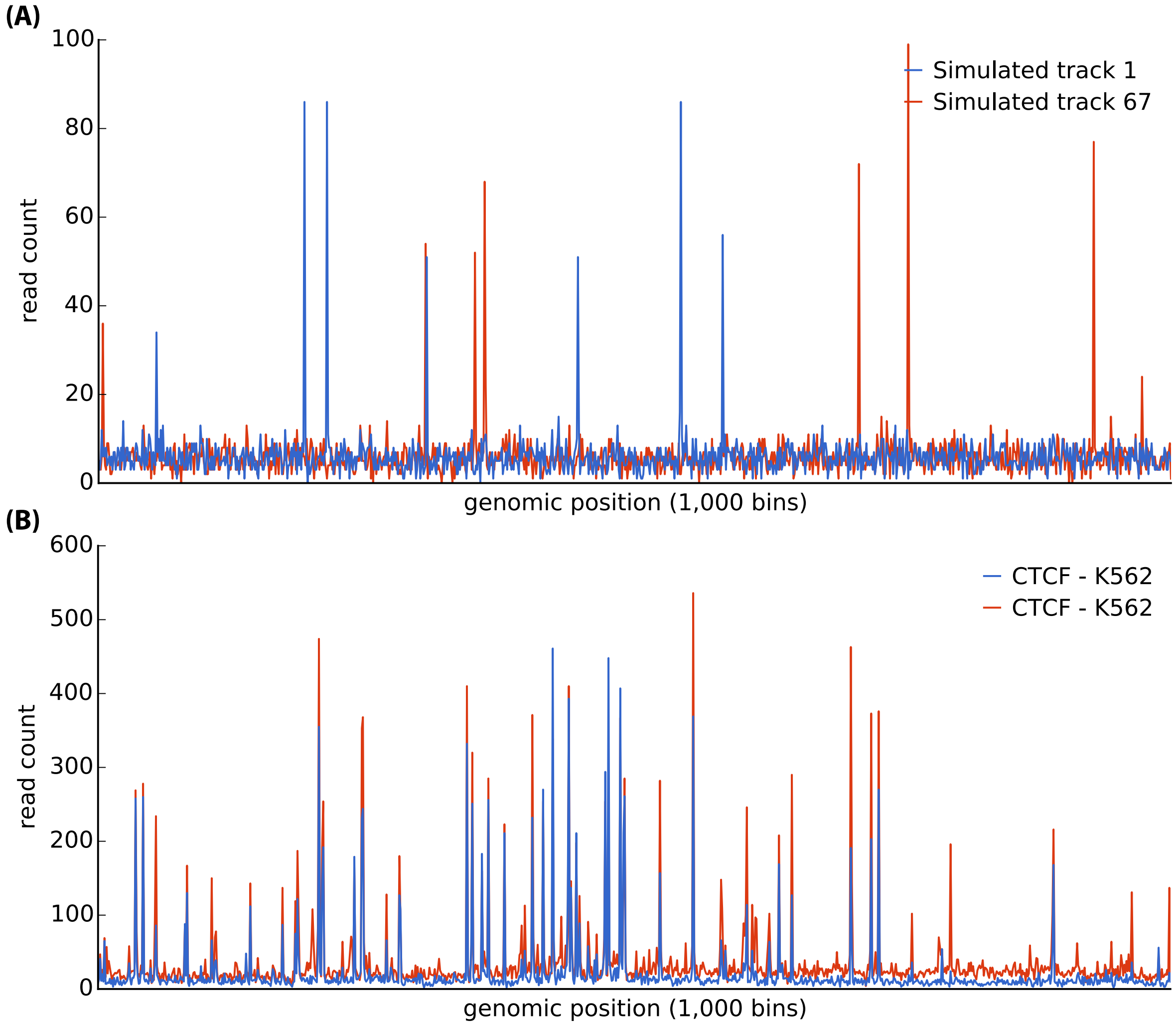

Figure S12: Visual comparison of simulated data and read data from two CTCF ChIP-seq tracks. While not identical, the simulated data is designed to be qualitatively similar to the distribution of real data tracks.

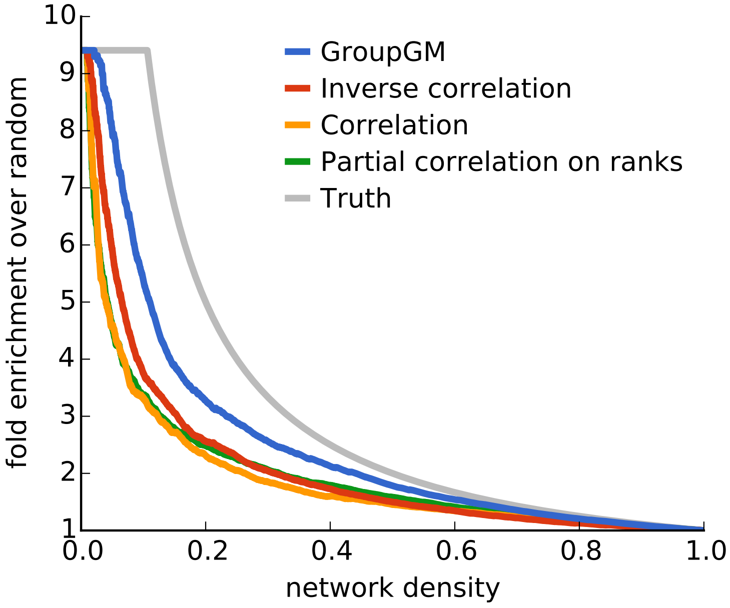

Figure S13: Results from a simulated data study with 126 datasets and 200,000 samples. Complexes of one, two and three simulated proteins were created where within complex correlations matched correlations observed in real data. Each method was then run and compared to known simulated interactions between complexes.

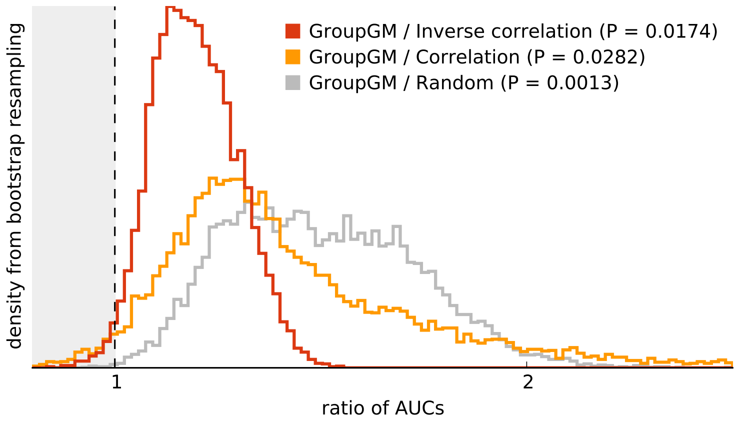

Figure S14: Histogram of area under the curve (AUC) ratios comparing enrichment of BioGRID-supported edges in a GroupGM network versus networks created by inverse correlation (red), correlation (yellow), and random assignment (grey). Specifically, we compared the area under enrichment–edge density curves from 10,000 bootstrap samples from regulatory factors, including edges between different cell types (Figure 3A bottom). Variability was higher than in an examination of edges within cell types (Figure S10). This is because resampling regulatory factors measured in many cell types alters many edges across cell types.

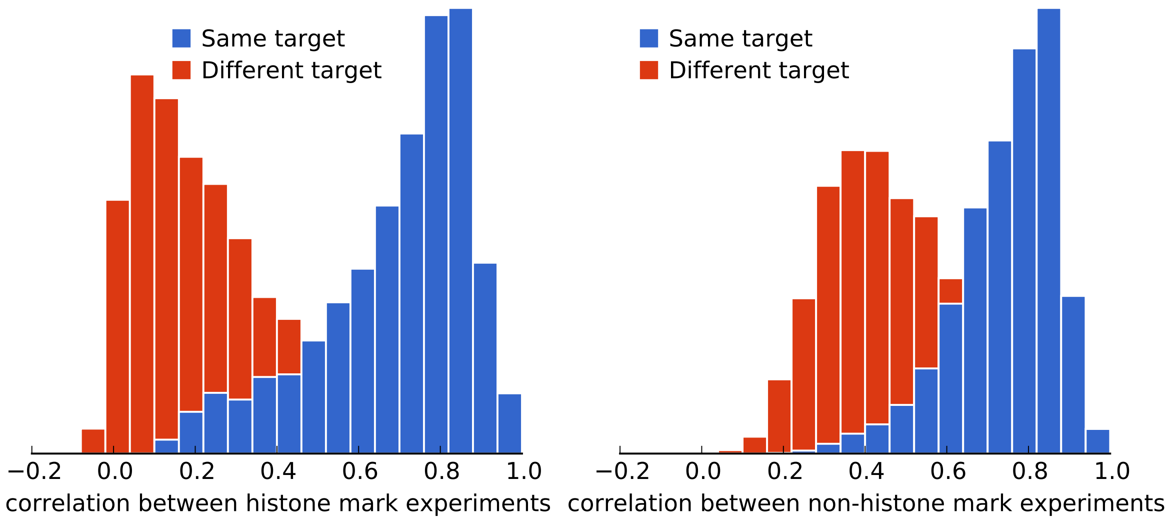

Figure S15: Correlations of the same factor between different cell types. Correlations between the same histone marks in different cell types is shown on the left, while correlations between the same non-histone factors is shown on the right. The clear bias towards positive correlation is likely the result of regions of consistent chromatin accessibility and mappability between all ChIP-seq datasets.

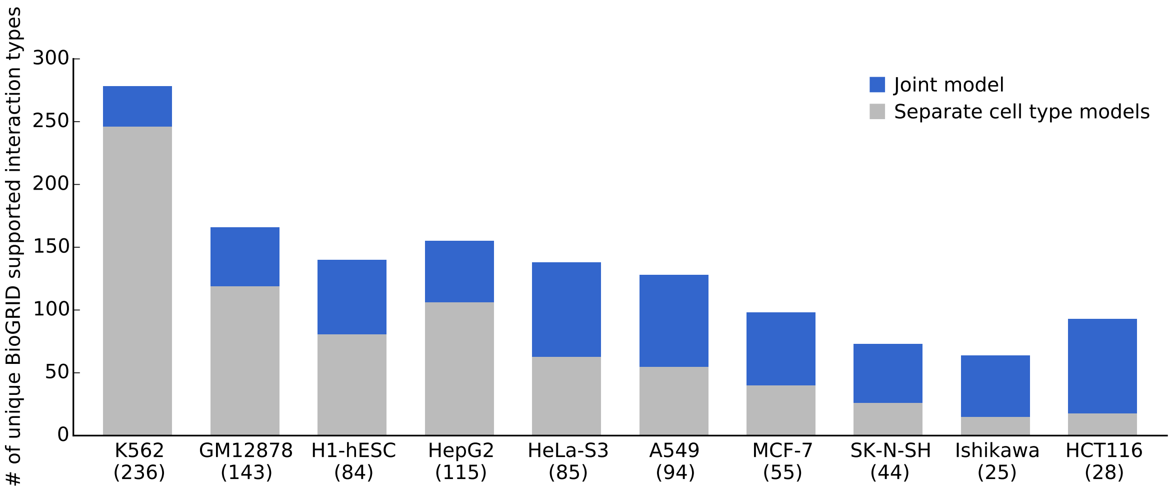

Figure S16: A joint model allows comparison with datasets not only within a single cell type but also across cell types. Here the increased number of BioGRID supported unique factor-factor interaction types detected at a threshold of 0.2 by a joint model is shown for each cell type.

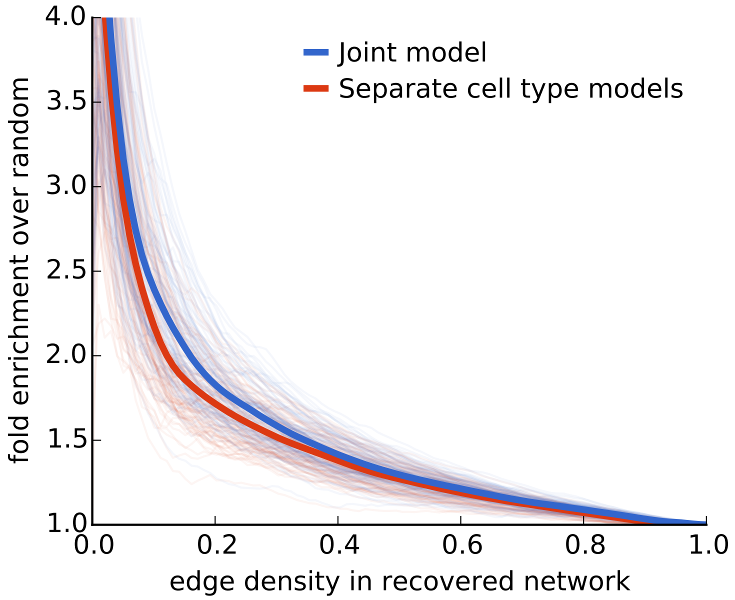

Figure S17: Comparison of a joint GroupGM of all cell types vs. individually learned GroupGM networks for each cell type. cross-cell-type edges from the joint model are ignored and only edges common to both networks are compared for enrichment of BioGRID supported edges. The joint model is marginally better than individual cell type models (P-value = 0.0672).

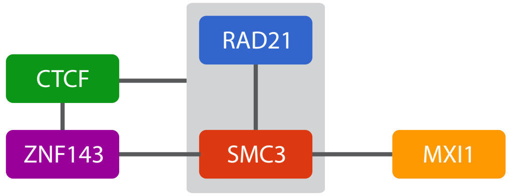

Figure S18: Similar to Figure 4A but with a CTCF experiment and ZNF143 experiment added to the figure. The network edges are from a GroupGM model with an edge threshold of 0.3. Both new experiments tend to associate with the cohesion complex proteins RAD21 and SMC3. The CTCF association is consistent with its combined role with cohesion in mediating chromosomal structure [9].

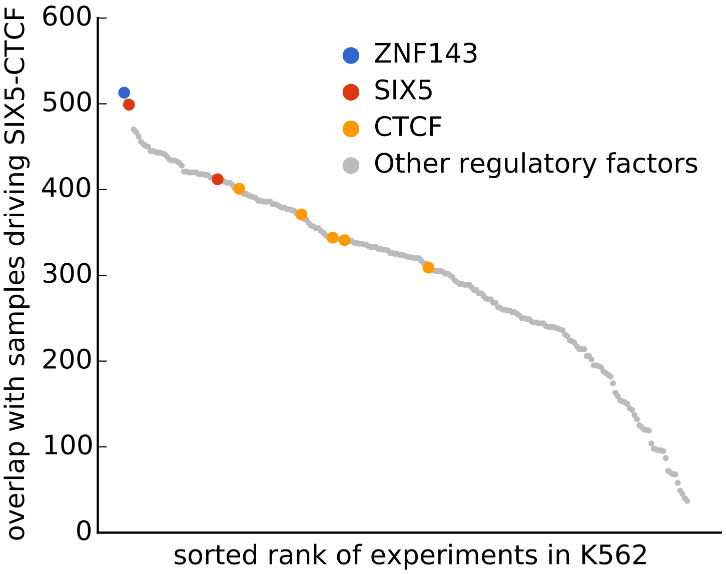

Figure S19: If all ZNF143 datasets from ChromNet are removed and then samples that drive a connection between CTCF and SIX5 in K562 are estimated we find that those samples strongly overlap with positions where ZNF143 is present. The top 1,000 positions driving the edge between SIX5 and CTCF overlap more strongly with the highest 1,000 ZNF143 positions than with any other dataset in K562, including the CTCF and SIX5 datasets the edge actually connects.

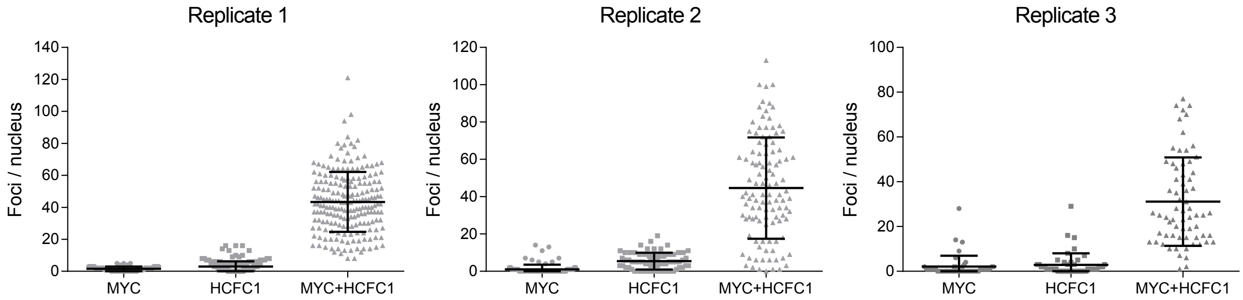

Figure S20: Quantifications for each independent replicate for the MYC–HCFC1 proximity ligation assay. Signal is quantified as the number of foci per nucleus. Individual values (grey dots) and mean ± standard deviation black bars are shown for each replicate.

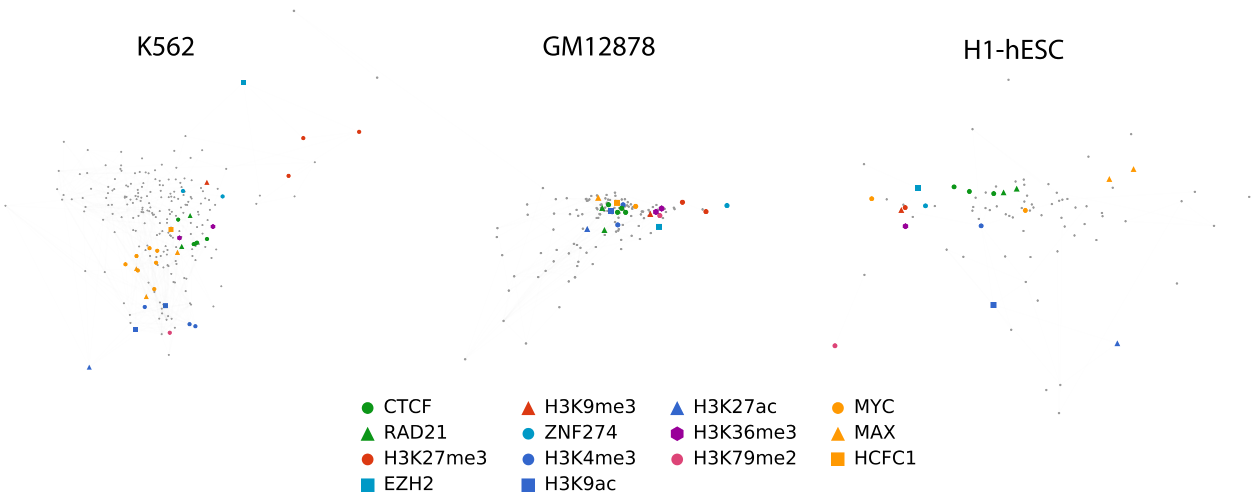

Figure S21: Embeddings of cell-type specific networks using the same approach as in Figure 6 (Methods). All three Tier 1 ENCODE cell types are highlighted with the same coloring used in Figure 6A.

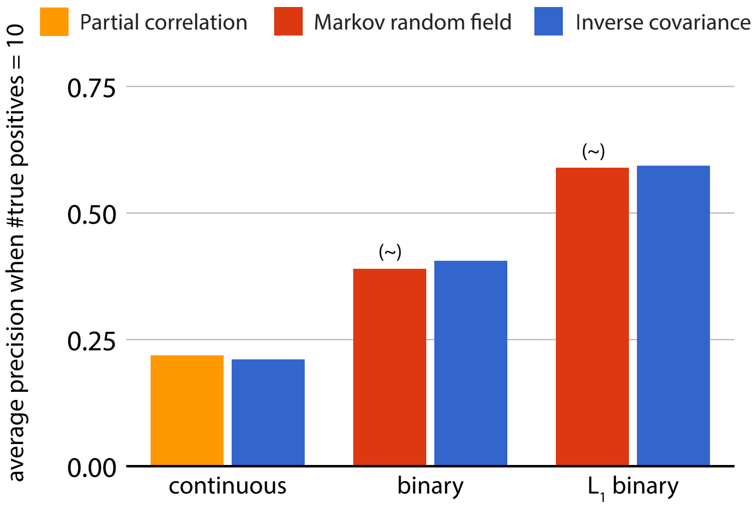

Figure S22: Precision when predicting BioGRID interactions using inverse covariance (blue), a binary Markov random field model from [14] (red), and partial correlation (yellow). A tilde (˜) indicates we took Markov random field precision numbers directly from the published precision-recall plot in [14]. To generate inverse covariance and partial correlation results, we started with processed data from [14]. Then, we calculated bootstrap-averaged performance on BioGRID interactions as Zhou et al. did in their article. We compared methods under three different testing regimes. Continuous represents testing on the original control-adjusted, normalized, and binned data. Binary represents testing on binarized data, without regularization. L_1 binary represents testing on binarized data, with L_1 regularization of both models.

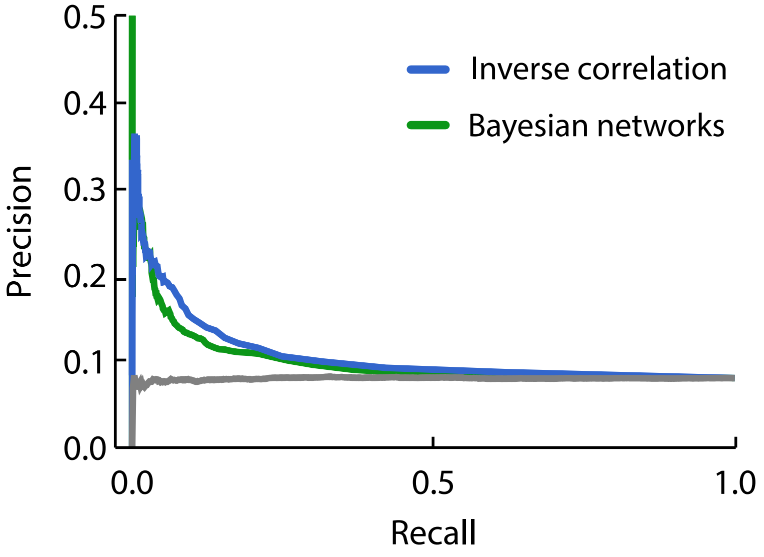

Figure S23: A precision-recall curve for known protein-protein interactions in BioGRID among experiments from the K562 cell type. Bootstrapped Bayesian network inference was performed as in previous work on *D. melanogaster* [11, 1]. We used networks from 400 bootstrap re-samples to estimate 400 Bayesian networks.

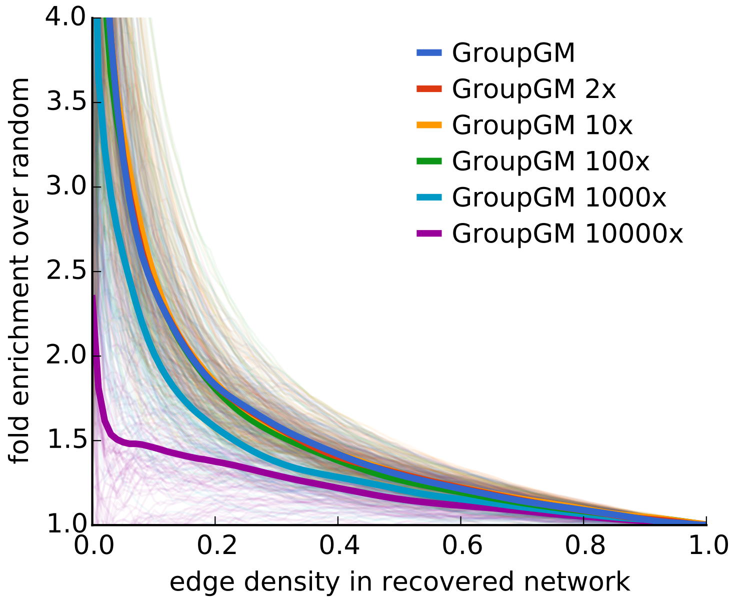

Figure S24: Enrichment of BioGRID supported edges within all cell types as the number of samples used to build the network is varied. Subsampling is done uniformly from the 1,000 bp bins across the genome and has the effect of both reducing the number of samples and also decreasing the correlation between neighboring samples. Up to 100-fold subsampling is possible before noticeable performance degradation.

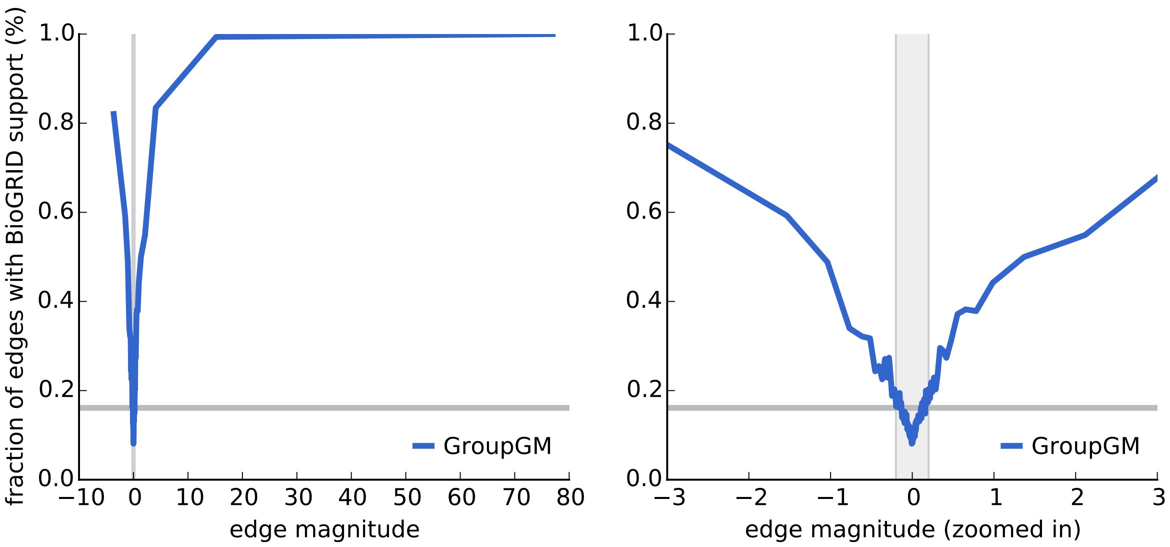

Figure S25: Enrichment of BioGRID support in edges with a given weight. Negative coefficients indicate negative correlation. Dark grey line indicates the fraction of BioGRID-supported edges in a randomly connected network (8.4%). Light grey shaded area represents those edges with coefficient magnitude less than the 0.2 minimum used in the ChromNet interface.

Supplementary tables

Table S1: Summary of all ENCODE datasets processed by ChromNet broken down by cell type. This summarizes the full listing of all 1,451 datasets with ENCODE experiment identifiers (Supplementary Data 1). The transcription factor and histone columns represent how many unique transcription factors or histone modifications were measured in that cell type. The treatments column lists the number of additional treatment conditions each cell type was measured under.

Cell type	Datasets	Transcription factors	Histone modifications	Treatments
**K562**	236	154	12	2
**GM12878**	143	107	11	1
**HepG2**	115	81	11	3
**A549**	94	51	11	2
**HeLa-S3**	85	62	11	1
**H1-hESC**	84	60	11	0
**MCF-7**	55	35	6	1
**SK-N-SH**	44	27	6	1
**endothelial cell of umbilical vein**	28	9	12	0
**HCT116**	28	22	5	0
**Ishikawa**	25	21	0	6
**fibroblast of lung**	22	2	11	0
**keratinocyte**	19	2	12	0
**neural cell**	17	9	8	0
**mammary epithelial cell**	16	2	11	0
**SUDHL6**	14	2	12	0
**Karpas-422**	14	2	12	0
**CD14-positive monocyte**	14	1	11	0
**skeletal muscle myoblast**	13	2	11	0
**myotube**	13	2	11	0
**fibroblast of dermis**	13	2	11	0
**astrocyte**	13	2	11	0
**Panc1**	13	4	6	0
**DND-41**	13	2	11	0
**osteoblast**	12	2	10	0
**cardiac mesoderm**	12	0	3	0
**MCF 10A**	12	5	0	1
**HEK293**	12	7	5	0
**DOHH2**	12	1	11	0
**OCI-LY7**	11	1	10	0
**OCI-LY3**	11	1	10	0
**OCI-LY1**	11	0	11	0
**Loucy**	11	1	10	0
**IMR-90**	10	10	0	0
**GM12891**	10	9	0	1
**T47D**	9	6	0	4
**NT2/D1**	9	3	6	0

Cell type	Datasets	Transcription factors	Histone modifications	Treatments
**GM12892**	8	7	0	1
**B cell**	8	2	5	0
**PFSK-1**	6	5	0	0
**HL-60**	6	5	1	0
**NB4**	5	4	1	0
**mononuclear cell**	4	0	4	0
**kidney epithelial cell**	4	1	3	0
**foreskin fibroblast**	4	1	1	0
**bronchial epithelial cell**	4	1	3	0
**U2OS**	4	2	2	0
**GM06990**	4	1	3	0
**Caco-2**	4	1	3	0
**BJ**	4	1	3	0
**ACC112**	4	0	4	0
**erythroblast**	3	2	0	0
**cardiac fibroblast**	3	1	1	0
**WI38**	3	1	1	1
**SK-N-MC**	3	2	1	0
**LNCaP clone FGC**	3	1	1	1
**H7-hESC**	3	0	3	0
**retinal pigment epithelial cell**	2	1	1	0
**fibroblast of villous mesenchyme**	2	1	1	0
**fibroblast of upper leg skin**	2	1	1	0
**fibroblast of the aortic adventitia**	2	1	1	0
**fibroblast of skin of abdomen**	2	1	1	0
**fibroblast of pulmonary artery**	2	1	1	0
**fibroblast of pedal digit skin**	2	1	1	0
**fibroblast of mammary gland**	2	1	1	0
**fibroblast of gingiva**	2	1	1	0
**epithelial cell of proximal tubule**	2	1	1	0
**epithelial cell of esophagus**	2	1	1	0
**choroid plexus epithelial cell**	2	1	1	0
**cardiac muscle cell**	2	1	1	0
**brain microvascular endothelial cell**	2	1	1	0
**astrocyte of the spinal cord**	2	1	1	0
**astrocyte of the cerebellum**	2	1	1	0
**WERI-Rb-1**	2	1	1	0
**SH-SY5Y**	2	2	0	0
**HFF-Myc**	2	1	1	0
**H54**	2	2	0	0
**GM19193**	2	2	0	1
**GM19099**	2	2	0	1
**GM18951**	2	2	0	1
**GM18526**	2	2	0	1
**GM18505**	2	2	0	1

Cell type	Datasets	Transcription factors	Histone modifications	Treatments
**GM15510**	2	2	0	1
**GM12875**	2	1	1	0
**GM12866**	2	1	1	0
**GM12865**	2	1	1	0
**GM12864**	2	1	1	0
**GM10847**	2	2	0	1
**GM08714**	2	1	1	0
**BE2C**	2	1	1	0
**spleen**	1	1	0	0
**skeletal muscle cell**	1	0	1	0
**pancreas**	1	1	0	0
**medulloblastoma**	1	1	0	0
**lung**	1	1	0	0
**kidney**	1	1	0	0
**Raji**	1	1	0	0
**Jurkat**	1	0	1	0
**GM20000**	1	1	0	0
**GM19240**	1	1	0	0
**GM19239**	1	1	0	0
**GM19238**	1	1	0	0
**GM13977**	1	1	0	0
**GM13976**	1	1	0	0
**GM12874**	1	1	0	0
**GM12873**	1	1	0	0
**GM12872**	1	1	0	0
**GM12871**	1	1	0	0
**GM12870**	1	1	0	0
**GM12869**	1	1	0	0
**GM12868**	1	1	0	0
**GM12867**	1	1	0	0
**GM12801**	1	1	0	0
**GM10266**	1	1	0	0
**GM10248**	1	1	0	0
**Total**	**1,451**	**812**	**376**	**33**

K562	GM12878	H1-hESC	HepG2	A549	HeLa-S3	SK-N-SH
POLR2A	POLR2A	POLR2A	POLR2A	POLR2A	POLR2A	REST
**EP300**	MTA3	TAF1	**EP300**	NR3C1	**EP300**	**EP300**
MAX	NFIC	**EP300**	NFIC	SP1	SMARCC1	POLR2A
MTA3	ATF2	GABPA	SP1	**EP300**	TBP	RAD21
WHSC1	YY1	HDAC2	MBD4	MAX	MAX	MXI1
eGFP-JUND	STAT5A	RBBP5	MAX	SIN3A	SREBF2	YY1
HDAC2	SP1	CHD1	FOXA2	FOSL2	CEBPB	RFX5
CBX3	IKZF1	CTBP2	TBP	REST	TAF1	JUND
YY1	RUNX3	ATF2	MYBL2	SIX5	MYC	SMC3
MYC	**EP300**	SP1	ZHX2	USF1	ELK4	CTCF

Table S2: H3K4me1 and H3K27ac combine to mark active enhancers [2]. Here group edges from these two histone marks are computed to all other non-histone regulatory factors and the top ten within each cell type are listed. The well known enhancer associated transcription factor EP300 [10] is found in each cell type and is a validation that we are finding enhancer associated transcription factors (P-value $< 1 \times 10^{-6}$). Using a false discovery threshold of 0.1 we highlighted in red those factors with a significant bias towards a high ranked associated with H3K4me1 and H3K27ac in these cell types.

Supplementary Note 1: Scalability of previous methods

Only correlation and inverse correlation are compared to ChromNet for the full human chromatin network in Figure 3. This is because the other previous methods we considered could not scale to the full 1,451 datasets. These are ARACNE (a well-known network learning method for gene expression data) [7], binary Markov random fields [14], and bootstrapped Bayesian networks [11, 1].

ARACNE is designed to handle gene expression which contains a large number of variables, but not necessarily a large number of samples. This was evident when we sought to apply it to chromatin network estimation. ARACNE exhausted all memory on a 24 gigabyte system with only 10 variables and 100,000 samples. This precludes it from even approaching the 3 million samples and 1,451 variables in the ENCODE dataset.

Binary Markov random fields were used successfully to recover regulatory factor interactions in *D. melanogaster*, with 73 variables and 100,000 samples [14]. Using the code kindly provided by Zhou et al., we attempted to apply the Markov random field to the human ENCODE data. Estimating the full joint distribution of a binary Markov random field model is very expensive. One approximation that is much more efficient involves the use of the psuedo-likelihood instead of the joint likelihood. This was one of the methods used by Zhou et al. [14], however even the pseudo-likelihood becomes intractable when we consider all ENCODE datasets, taking over 10 hours with just 60 variables in the model (Figure S5). Furthermore, when we compared inverse correlation to the Binary Markov random field recovery methods on the original *D. melanogaster* data we obtained equivalent performance (Figure S22).

Bootstrapped versions of Bayesian network inference have been used previously to infer networks among regulatory factors in *D. melanogaster* [11, 1]. These experiments were run on binary data among up to 112 factors, but scaling them to human data is much more challenging. Because of run-time constraints we restricted the model to only consider 238 datasets from the K562 cell type. We then used networks from 400 bootstrap re-samples to estimate 400 networks. Each network

Regulatory factor	Max total edge weight	Known in BioGRID
**MAX**	5.93	+
**POLR2A**	1.66	+
**PHF8**	1.26	−
**NEUROD1**	0.81	−
**CREB3L1**	0.79	−
**CEBPB**	0.78	+
**HCFC1**	0.73	−
**ATF7**	0.70	−
**SUZ12**	0.67	−
**EP300**	0.64	+

Table S3: Top 10 (out of 193) regulatory factors with a strong connection to MYC in ChromNet. Scores are the sum of within cell type group edges connecting MYC experiments to the listed factor. The maximum score is then taken over all ENCODE tier 1 cell types. For comparison we also ran the same experiment using standard correlation instead of group edges and HCFC1 was the 31st strongest interaction with MYC (as opposed to the 7th here).

took about 1.2 hours of processing time to find good solutions, leading to over 500 CPU hours of compute time. Inverse correlation uses a normal approximation for the binary data, runs in less than 10 seconds, and out-performs the far less efficient Bayesian network inference method in terms of known agreement with physical protein-protein interactions labeled in BioGRID (Figure S23).

Note that to allow for a direct comparison with these methods we used binary data rather than raw read counts. The inverse correlation model still outperformed or matched these methods even on their own data types. GroupGM provides and additional level of improvement on top of inverse correlation as demonstrated in Figure 3.

Supplementary Note 2: Proof that the group graphical model preserves edge magnitudes in the presence of arbitrary collinearity

The inverse covariance matrix (a symmetric matrix) can be interpreted in terms of multiple regression [5, 12], where for simplicity of notation we assume infinite data samples so $\hat{\Sigma} = \Sigma$:

$$\Sigma^{-1} = \Omega = \begin{bmatrix} 1/[\Sigma_{11}(1-R_1^2)] & -\beta_{12}/[\Sigma_{11}(1-R_1^2)] & \cdots & -\beta_{1n}/[\Sigma_{11}(1-R_1^2)] \\ -\beta_{21}/[\Sigma_{22}(1-R_2^2)] & 1/[\Sigma_{22}(1-R_2^2)] & \cdots & -\beta_{2n}/[\Sigma_{22}(1-R_2^2)] \\ \vdots & \vdots & \ddots & \vdots \\ -\beta_{n1}/[\Sigma_{nn}(1-R_n^2)] & -\beta_{n2}/[\Sigma_{nn}(1-R_n^2)] & \cdots & 1/[\Sigma_{nn}(1-R_n^2)] \end{bmatrix}$$

where β_{ij} is a parameter of the ith regression that predicts the ith variable from all the others, and R_i^2 is the proportion of the variance in variable i explained by the ith regression. For correlation matrices the on-diagonal Σ_{ii} entries will be one:

$$\Omega = \begin{bmatrix} 1/(1-R_1^2) & -\beta_{12}/(1-R_1^2) & \cdots & -\beta_{1n}/(1-R_1^2) \\ -\beta_{21}/(1-R_2^2) & 1/(1-R_2^2) & \cdots & -\beta_{2n}/(1-R_2^2) \\ \vdots & \vdots & \ddots & \vdots \\ -\beta_{n1}/(1-R_n^2) & -\beta_{n2}/(1-R_n^2) & \cdots & 1/(1-R_n^2) \end{bmatrix}$$

To further simplify, we can define $S_i = \frac{-1}{1-R_i^2}$:

$$\Omega = \begin{bmatrix} -S_1 & S_1\beta_{12} & \cdots & S_1\beta_{1n} \\ S_2\beta_{21} & -S_2 & \cdots & S_2\beta_{2n} \\ \vdots & \vdots & \ddots & \vdots \\ S_n\beta_{n1} & S_n\beta_{n2} & \cdots & -S_n \end{bmatrix}$$

Consider an arbitrary edge between two nodes A and B with that correspond to rows A_1 and B_1 in Ω. The strength of the connection in the symmetric matrix Ω is $S_{A_1}\beta_{A_1B_1} = S_{B_1}\beta_{B_1A_1}$.

Now consider a new data set with a superset of the variables in the original network represented by Ω. This new dataset, represented by $\Omega^{(2)}$, has a second B variable with index B_2. These two B variables (B_1 and B_2) are arbitrarily similar to one another but not identical, and the second variable bears no relationship to other variables in the network beyond what it gains by being similar to B_1. The regression problem for A_1 would be unstable, because B_1 and B_2 are highly correlated to each other, which makes it unclear how the weights should be distributed to these two predictor variables. However, the sum of the coefficients for the B group remains the same:

$$\beta^{(2)}_{A_1B_1} + \beta^{(2)}_{A_1B_2} = \beta_{A_1B_1},$$

In addition, no new information has been provided about A, so S_A remains unchanged (because the amount of variance explained remains the same):

$$S^{(2)}_A = S_A,$$

which means the following:

$$S^{(2)}_A\beta^{(2)}_{A_1B_1} + S^{(2)}_A\beta^{(2)}_{A_1B_2} = S_A\beta_{A_1B_1},$$

which is equivalent to:

$$\Omega^{(2)}_{A_1B_1} + \Omega^{(2)}_{A_1B_2} = \Omega_{A_1B_1}.$$

This means that the connection strength that was present in between A and B in Ω is now preserved as a sum of two entries in $\Omega^{(2)}$. This argument generalizes to any number of variables in the B group.

Now after adding a redundant B variable consider adding a redundant A variable to create a new data set $\Omega^{(3)}$. Since the B variables cannot choose between A_1 and A_2 their coefficients are unstable but still sum to their previous value:

$$\beta^{(3)}_{B_1A_1} + \beta^{(3)}_{B_1A_2} = \beta^{(2)}_{B_1A_1} \quad (1)$$

$$\beta^{(3)}_{B_2A_1} + \beta^{(3)}_{B_2A_2} = \beta^{(2)}_{B_2A_1} \quad (2)$$

adding A_2 provided no new explanatory power for the B variables so

$$S^{(3)}_{B_1} = S^{(2)}_{B_1} \quad (3)$$

$$S^{(3)}_{B_2} = S^{(2)}_{B_2}, \quad (4)$$

which means

$$S^{(3)}_{B_1}\beta^{(3)}_{B_1A_1} + S^{(3)}_{B_1}\beta^{(3)}_{B_1A_2} = S^{(2)}_{B_1}\beta^{(2)}_{B_1A_1} \tag{5}$$
$$S^{(3)}_{B_2}\beta^{(3)}_{B_2A_1} + S^{(3)}_{B_2}\beta^{(3)}_{B_2A_2} = S^{(2)}_{B_2}\beta^{(2)}_{B_2A_1}, \tag{6}$$

and

$$\Omega^{(3)}_{B_1A_1} + \Omega^{(3)}_{B_1A_2} = \Omega^{(2)}_{B_1A_1} \tag{7}$$
$$\Omega^{(3)}_{B_2A_1} + \Omega^{(3)}_{B_2A_2} = \Omega^{(2)}_{B_2A_1}. \tag{8}$$

Because Ω is symmetric we know that

$$\Omega^{(2)}_{A_1B_1} + \Omega^{(2)}_{A_1B_2} = \Omega^{(2)}_{B_1A_1} + \Omega^{(2)}_{B_2A_1}.$$

Using this we can now calculate the original connection strength $\Omega_{A_1B_1}$ as a sum of entries in $\Omega^{(3)}$. This can be directly generalized to any number of variables in each group, which means that the connection strength of an edge between two variables in a non-redundant data set can be recovered by summing edges in a data set where both variables are in groups of redundant variables.

$$\Omega_{A_1B_1} = \Omega^{(2)}_{A_1B_1} + \Omega^{(2)}_{A_1B_2} \tag{9}$$
$$\Omega_{A_1B_1} = \Omega^{(2)}_{B_1A_1} + \Omega^{(2)}_{B_2A_1} \tag{10}$$
$$\Omega_{A_1B_1} = \Omega^{(3)}_{B_1A_1} + \Omega^{(3)}_{B_1A_2} + \Omega^{(3)}_{B_2A_1} + \Omega^{(3)}_{B_2A_2} \tag{11}$$
$$\tag{12}$$

Supplementary Note 3: Estimation of conditional dependence from binary data

For the binary data tracks compared in Figure S1 we matched datasets with controls using metadata from the ENCODE web site [3], then ran MACS2 [13] without peak shift adjustments and with a P-value peak threshold of 0.05. Peak data from MACS2 was then binned into 1,000 bp windows by labeling a window 1 if any peak overlapped the window and 0 otherwise.

Here we briefly discuss why we considered binary data in the context of inverse correlation. Given datasets drawn from a set $\mathcal{X}_b$ of binary random variables, we can represent a joint pairwise model of these datasets without loss of generality as a pairwise Markov random field:

$$P(x) = \frac{1}{Z}\exp\left(-\sum_{X_i\in\mathcal{X}_b, X_j\in\mathcal{X}_b} \Phi_{i,j}X_iX_j\right) \tag{13}$$

where Φ is a matrix of pairwise interaction terms and Z is a normalizing constant. Previous work on estimating a smaller subset of the chromatin network from binary data used Markov random fields and higher-order extensions [14, 8]. These works employ iterative or approximate methods, as exact inference on their models with many variables is computationally intractable. For certain graph classes, however, the sparsity structure of Φ and of the inverse correlation matrix Σ^{-1} are equivalent [6].

To compare Σ^{-1} with one of these methods we compared with estimates of conditional dependence from a pairwise Markov random field of binary data [14] (Supplementary Note 2). The

Markov random field implementation was based on unnormalized binary data, so for comparison we used the inverse covariance matrix which results from an unnormalized dataset (meaning the data was not mean centered or scaled to unit variance). We used the original processed data kindly provided by the authors of [14] (J. Zhou, personal communication). These data are from 73 modENCODE ChIP-chip datasets on *Drosophila melanogaster* S2-DRSC cells. We calculated precision for the inverse covariance of binary data using the same bootstrap procedure as the authors, and compared against Markov random field precision numbers from their published precision-recall plot [14].

On this smaller data set, the enrichment of known protein-protein interactions in $\hat{\Phi}$ and the inverse covariance matrix were similar (Figure S22). This near-equivalence between the methods supports the use of an inverse covariance (or correlation) matrix to estimate edge strength in a pairwise Markov random field. It is infeasible to estimate a Markov random field among all ENCODE datasets (Supplementary Note 1). Using a matrix inverse dramatically increases computational efficiency, while maintaining results similar to a full binary pairwise Markov random field.

References

[1] Joke G van Bemmel et al. "A network model of the molecular organization of chromatin in Drosophila". In: *Molecular cell* 49.4 (2013), pp. 759–771.

[2] Menno P Creyghton et al. "Histone H3K27ac separates active from poised enhancers and predicts developmental state". In: *Proceedings of the National Academy of Sciences* 107.50 (2010), pp. 21931–21936.

[3] *Encode Project Data*. encodeproject.org.

[4] Satyajeet P Khare et al. "HIstome—a relational knowledgebase of human histone proteins and histone modifying enzymes". In: *Nucleic acids research* 40.D1 (2012), pp. D337–D342.

[5] Clarence CY Kwan. "A Regression-Based Interpretation of the Inverse of the Sample Covariance Matrix". In: *Spreadsheets in Education (eJSiE)* 7.1 (2014), p. 3.

[6] Po-Ling Loh, Martin J Wainwright, et al. "Structure estimation for discrete graphical models: Generalized covariance matrices and their inverses". In: *The Annals of Statistics* 41.6 (2013), pp. 3022–3049.

[7] Adam A Margolin et al. "ARACNE: an algorithm for the reconstruction of gene regulatory networks in a mammalian cellular context". In: *BMC bioinformatics* 7.Suppl 1 (2006), S7.

[8] Martin Renqiang Min et al. "Interpretable sparse high-order boltzmann machines". In: *Proceedings of the Seventeenth International Conference on Artificial Intelligence and Statistics.* 2014, pp. 614–622.

[9] Vania Parelho et al. "Cohesins functionally associate with CTCF on mammalian chromosome arms". In: *Cell* 132.3 (2008), pp. 422–433.

[10] Daria Shlyueva, Gerald Stampfel, and Alexander Stark. "Transcriptional enhancers: from properties to genome-wide predictions". In: *Nature Reviews Genetics* 15.4 (2014), pp. 272–286.

[11] Bas van Steensel et al. "Bayesian network analysis of targeting interactions in chromatin". In: *Genome research* 20.2 (2010), pp. 190–200.

[12] Guy VG Stevens. "On the inverse of the covariance matrix in portfolio analysis". In: *The Journal of Finance* 53.5 (1998), pp. 1821–1827.

[13] Yong Zhang et al. "Model-based analysis of ChIP-Seq (MACS)". In: *Genome Biology* 9.9 (2008), R137.

[14] Jian Zhou and Olga G Troyanskaya. "Global quantitative modeling of chromatin factor interactions". In: *PLoS Computational Biology* 10.3 (2014), e1003525.
